# Study on an Agricultural Environment Monitoring Server System using Wireless Sensor Networks

**DOI:** 10.3390/s101211189

**Published:** 2010-12-08

**Authors:** Jeonghwan Hwang, Changsun Shin, Hyun Yoe

**Affiliations:** School of Information and Communication Engineering, Sunchon National University, Maegok-dong, Suncheon-si, Jeollanam-do, Korea; E-Mails: jhwang@sunchon.ac.kr (J.H.); csshin@sunchon.ac.kr (C.S.)

**Keywords:** ubiquitous, agriculture, wireless sensor networks, monitoring server

## Abstract

This paper proposes an agricultural environment monitoring server system for monitoring information concerning an outdoors agricultural production environment utilizing Wireless Sensor Network (WSN) technology. The proposed agricultural environment monitoring server system collects environmental and soil information on the outdoors through WSN-based environmental and soil sensors, collects image information through CCTVs, and collects location information using GPS modules. This collected information is converted into a database through the agricultural environment monitoring server consisting of a sensor manager, which manages information collected from the WSN sensors, an image information manager, which manages image information collected from CCTVs, and a GPS manager, which processes location information of the agricultural environment monitoring server system, and provides it to producers. In addition, a solar cell-based power supply is implemented for the server system so that it could be used in agricultural environments with insufficient power infrastructure. This agricultural environment monitoring server system could even monitor the environmental information on the outdoors remotely, and it could be expected that the use of such a system could contribute to increasing crop yields and improving quality in the agricultural field by supporting the decision making of crop producers through analysis of the collected information.

## 1. Introduction

Recently, innovations in information communication technology have been accelerating the convergence between different industries [1,2]. The convergence and integration of IT with agricultural technology is expected to be an area that could increase the added value and productivity of agriculture by applying the ubiquitous technology to the agricultural sector which is a primary industry [3,4].

To successfully construct such a u-agricultural environment, the development of essential ubiquitous technology optimized for agriculture such as sensor hardware, middleware platforms, routing protocols and application services for agricultural environments is needed.

Examples of the convergence of ubiquitous technology with agriculture, which is a primary industry, on a trial basis exist, such as the use of sensor nodes in vine culture sites and applications of ubiquitous technology in livestock farming sites, and the technology has gradually begun to appear in other small areas like the increase of production and the improvement of quality at various agricultural sites [5–9].

An agricultural environment monitoring system provides environmental monitoring services and facility controlling services, and thus maintains the crop growing environment in an optimal status. This system also improves the convenience and productivity of users. However, existing agricultural monitoring systems are mostly applied and utilized in closed agricultural environments such as greenhouses, cattle sheds, *etc.*, as it is difficult to apply agricultural monitoring systems in outdoors locations such as paddies, fields, orchards, *etc.* because of a lack of IT infrastructure. In addition, when users want to verify the monitored information in existing monitoring systems, the user must manually check the status through installed sensors or terminals installed in the agriculture facilities.

In order to solve these problems, it is necessary to develop an agricultural environment monitoring system that can monitor environmental information and soil information in remote location and can be used in agricultural environments which lack infrastructure. This paper proposes an agricultural environment monitoring server system to monitor information on the outdoors by utilizing WSN (wireless sensor network) technology, which is one of such ubiquitous technologies.

If the proposed agricultural environment monitoring server system is applied to an agricultural environment, environmental and soil information could be monitored even at a remote site, and it is expected that this would contribute to increased crop yields and the improvement of quality in the agricultural field by supporting producers’ decision making about crop production through the analysis on the collected information.

The agricultural environment monitoring server system proposed in this paper collects environmental information such as luminance, temperature, humidity, wind direction, wind speed, EC, pH, CO_2_ *etc.* which affect growth of crops and soil information through the WSN environmental sensors and soil sensors installed outdoors, collects image information on the outdoors and location information on the position where the server system is installed through CCTVs and GPS modules, and the information is converted into a database through the agricultural environment monitoring server to provide suitable information to producers through to a variety of services. In addition, the server system is set up to use power supplied through solar cells so that it could be used in agricultural environments with insufficient power infrastructure.

This paper is organized as follows. Section 2 describes related research, Section 3 introduces the structure and components of the system proposed in this paper and the services provided by the system, Section 4 describes in detail the functions of the system, construction methods for each module, performance measurements for each module, implementation and performance measurements of the final system, and Section 5 presents our conclusions.

## 2. Related Research

### 2.1. Environmental Monitoring

WSNs have become an important tool in environmental monitoring. The relatively low cost of the devices allows the installation of a dense population of nodes that can adequately represent the variability present in the environment. They can provide risk assessment information, like for example alerting farmers at the onset of frost damage and providing better microclimate awareness.

Johnson and Margalho monitored the agroclimate in the Amazon, analyzing short range WSN transmissions. They found that more distant nodes suffered a performance loss, while nodes closer to the sink maintained their throughput levels.

Another example of climate supervision is flood prediction by means of wireless sensors which can detect rainfall, water levels and weather conditions. The sensors supply information to a centralized database system [10]. Pierce and Elliot extended the implementation to a regional and on-farm sensor network operating at 900 MHz that provides remote, real-time monitoring and control of farming operations in two agricultural applications, a weather monitoring network and an on-farm frost monitoring network [11].

Ayday and Safak presented a moisture distribution map obtained through the integration of a WSN with a GIS (Geographic Information System). The wireless nodes with moisture sensors were located at predetermined locations; geographic coordinates of these points were obtained with GPS and then, all the information was evaluated using the GIS [12].

Han *et al.* developed a wireless data transmission system using wireless ZigBee motes, developed to remotely monitor in real time sediment runoff at a low-water crossings. The gateway transmitted the sensor signals to an Internet server using the GPRS [13].

Hamrita and Hoffacker developed a lab prototype for wireless measurement of soil temperatures. The system was based on a commercial 13.56 MHz RFID tag. Measurements showed a high correlation (greater than 99%) with those obtained using a thermocouple [14].

### 2.2. Precision Agriculture

The development of WSN applications in precision agriculture makes it possible to increase efficiencies, productivity and profitability in many agricultural production systems, while minimizing unintended impacts on wildlife and the environment. The real time information obtained from the fields can provide a solid base for farmers to adjust strategies at any time. Instead of making decisions based in some hypothetical average condition, which may not exist anywhere in reality, a precision farming approach recognizes differences and adjusts management actions accordingly [15].

Baggio deployed a WSN for fighting phytophtora in a potato field. Phytophtora is a fungal disease which depends on the climatological conditions. 868/916 MHz motes were used for measuring humidity and temperature. The aim of the system was to reveal when the crop was at risk and let the farmer treat the plants only when really needed [16].

### 2.3. Viticulture

Plant monitoring, also called phytomonitoring, is easier using WSNs. For example, with the help of a WSN the owner of vineyard can manage the operation of the vineyard more efficiently and automatically. Burrell *et al.* have described a variety of sensor network configurations and applications that can address different priorities in the vineyard [17]. Beckwith *et al.* implemented a WSN in a vineyard consisting of 65,916 MHz motes. Temperature measurements were collected during one month. The information was used for addressing two important parameters in wine production: heat accumulation and potential frost damage [18].

Morais *et al.* have shown the feasibility of a ZigBee based remote sensing network, intended for precision viticulture. The network nodes were powered by batteries that are recharged with energy harvested from the environment [19].

### 2.4. Greenhouses

Automation and efficiency are crucial in greenhouse environment monitoring and control. In order to control and monitor the environmental factors, sensors and actuators are essential. Greenhouse crops can benefit a lot from the use of WSNs, because inside the greenhouse the crop conditions such as climate and soil do not depend on exterior factors and the implementations are thus easier than in outdoor applications.

The first application of WSN in a greenhouse was reported in the year 2003. It was a monitoring and control system developed by means of Bluetooth [20]. Since then, several applications have been developed, most of them making use of IEEE 802.15.4/ZigBee. For example, Gonda and Cugnasca presented a proposal of a distributed greenhouse control and monitoring system using ZigBee [21]. Yoo *et al.* described the results of real deployment of a WSN IEEE 802.15.4 compliant system to monitor and control the environment in greenhouses where melons and cabbages were being grown [22]. Lea-Cox *et al.* developed a WSN in a greenhouse, that integrates a variety of sensors which can measure substrate water, temperature, electrical conductivity, daily photosynthetic radiation and leaf wetness in real-time. Benefits came from an improved plant growth, more efficient water and fertilizer applications, together with a reduction in disease problems related to over-watering [23].

Liu *et al.* reported a WSN prototype with a two-part framework for greenhouses. In the first part, several sensor nodes were used to measure temperature, light and soil moisture. The other part consists of a GSM module and the management software based on a database running on the remote PC [24].

Zhou *et al.* designed a monitoring system based on ZigBee, using a star network topology inside the greenhouse and a mesh topology for the connection between the greenhouses and the management system [25]. Yang *et al.* reported a multi-functional remote sensing system that integrates RFID technology with spectral imaging and environmental sensing in a greenhouse. The multi-spectral imaging system was used for remote sensing of the canopy of cabbage seedlings. Greenhouse temperature, relative humidity, and lighting conditions were measured above the crop [26].

## 3. System Architecture and Service Process

The agricultural environment monitoring server system proposed in this paper collects environmental information such as luminance, temperature, humidity, wind direction, wind speed, EC, pH, CO_2_ *etc.* which affect growth of crops and soil information through the WSN environmental sensors and soil sensors installed at the outdoors, and collects image information on the outdoors and location information on the position where the server system is installed through CCTVs and GPS modules. The information collected in real time like this is converted into a database through the agricultural environment monitoring server, which provides suitable information to producers according to a variety of services. This section describes the structure and components of the proposed system and services provided by the system.

### 3.1. System Architecture

Existing web-based monitoring systems such as WAGRIT have a structure that separates data acquisition devices and the web server. However, the proposed agricultural environment monitoring server system has a structure that integrates the WSN sensors, CCTVs, GPS modules, database server, web server, DVR server, *etc.* to collect environmental information on the outdoors and image information into a device for collecting various pieces of information on the outdoors environment, and provides real-time monitoring and various application services based on this information.

**Figure 1.**
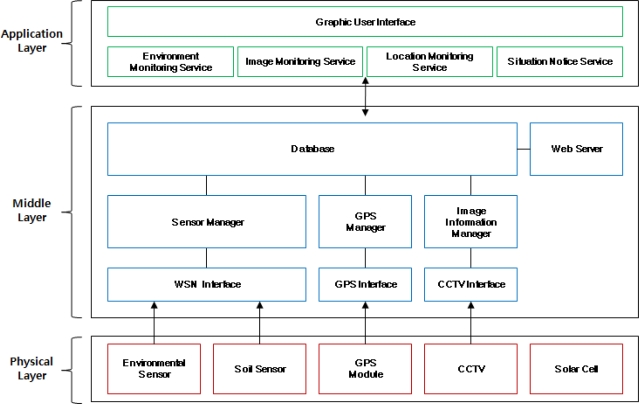
Agricultural Environment Monitoring Server System Architecture.

As shown in Figure 1, the proposed system is composed of a physical layer, which consists of WSN sensors, CCTV, GPS, solar cells *etc.* to collect information on the outdoors environment, a middle layer, which supports communications between the physical layer and the application layer and converts the outdoors information collected from the physical layer into a database to provide data requested from the application layer, and an application layer, which is equipped with interfaces to support various services for producers.

The WSN sensors are composed of environmental sensors to collect environmental information including luminance, humidity, temperature *etc.* and soil sensors to collect soil information including temperature, EC, soil pH, *etc*. After they are distributed outdoors and every sensor node forms an autonomous network, they send physical information acquired from sensor nodes to the server system by wireless.

The CCTV system collects image information on the outdoors in real time to provide outdoor images to user, and the GPS module collects location information on the installed server system and the collected outdoors information.

The solar cell supplies power for the server system installed outdoors, and the system is therefore applicable in an agricultural environment with insufficient power infrastructure because can operate even if no external power is supplied to the server system.

The sensor manager manages data acquisition from the soil and environmental sensors, extracts the soil and environmental data by processing the collected data packets into a format which could be stored in the database, and stores it in the database or sends it to other server systems for processing by converting the processed data into the format suitable for the measurement elements.

In addition, the expandability and convenience of the server system is improved by enabling it to select the sensors to be used and the database in which the data is to be stored through a simple menu in order to integrate and manage different kinds of sensors.

The GPS manager extracts location data coming in through serial communication from the GPS module to store it in the database, extracts the latitudes and longitudes from the received data using the &GPRMC format of NMEA 0183, and stores them in the database through an ‘Insert’ query statement after converting them appropriately for each unit.

The image information manager provides images taken from CCTVs to the Web as streaming data and stores them in the database.

The database stores environmental and soil data, image data, location information data and environmental reference values for notifying conditions into each table, and creates average statistical information by using the collected information.

The web server provides an environment that users could monitor data processed by the components and environment of the outdoors through a Web browser anywhere and at any time.

### 3.2. System Services

#### 3.2.1. Environmental Monitoring Service

The environmental monitoring service is a service to store in the database environmental and soil information collected from the WSN sensors installed on the outdoors, and allow users to monitor environmental information about the planted crops via the Web server anywhere in real time.

Looking at the detailed operation of this service, the WSN sensors installed in the outdoors collect environmental and soil information and periodically sent the sensing data to the sensor manager, then the sensor manager parses and analyzes the received sensing data to extract each sensing value and convert its format to store it into each table of the database.

**Figure 2.**
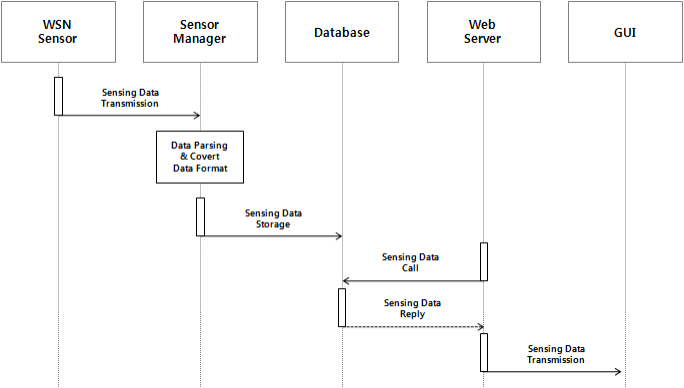
Operation Process of the Environment Monitoring Service.

The web server fetches environmental and soil information on the outdoors stored in the database to send it to a GUI at regular intervals, and users can monitor the outdoors environment information on via the GUI. Figure 2 shows the operating process of the environment monitoring service.

#### 3.2.2. Image Monitoring Service

**Figure 3.**
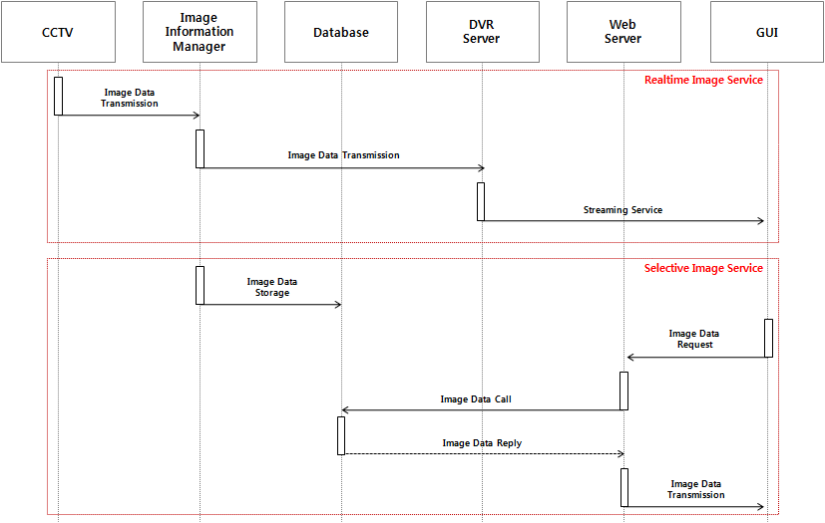
Operation Process of the Image Monitoring Service.

The image monitoring service is a service to provide image information on the outdoors to users through the CCTV installed on the server system. The CCTV sends the collected images on the outdoors to the image information manager, and the image information manager provides the received images to a GUI by means of real-time streaming service via a DVR server and classifies the streamed data to store it in the database. In addition, when users request image data stored in the database through the GUI, it would be provided to them. Figure 3 shows the operating process of the image monitoring service.

#### 3.2.3. Location Monitoring Service

**Figure 4.**
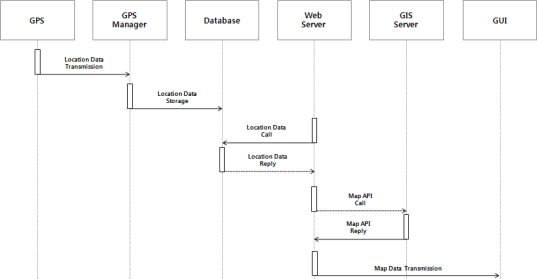
Operation Process of the Location Monitoring Service.

The location monitoring service is a service to provide location information on the server system and location information on the current monitoring place through the GPS module. The provision of location information is important because remote sites check for such information. The location monitoring service extracts latitude and longitude data from the location data received via the GPS module by the location information manager, and stores it into the database. Then, the Web server requests latitude and longitude data stored in the database to be sent it, and displays the location of the server system on a map based on the latitude and longitude data received through the GUI. Figure 4 shows the operating process of the location monitoring service.

#### 3.2.4. Condition Notification Service

**Figure 5.**
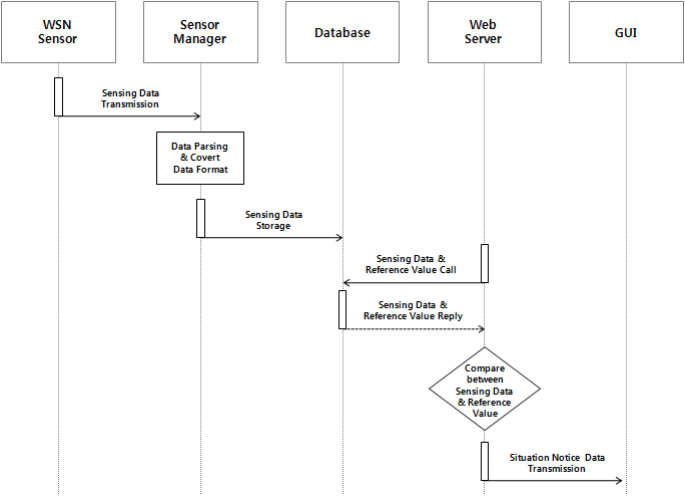
Operation Process of the Condition Notification Service.

The condition notification service is a service to prevent dangerous conditions beforehand by notifying users in real time about changes in the outdoors environment and taking some measure. Figure 5 shows the operating process of the condition notification service.

The data sensed by the environmental sensors is sent to the sensor manager. The sensor manager extracts sensing values from the received data to store them into the database. The Web server monitors the stored sensing values periodically to notify users that an event is ocurred if they are over or below some set reference value.

#### 3.2.5. Average Statistics Service

The average statistics service is a service to show users the sensing information incoming through environmental and soil sensors as a graphic form by compiling statistics into some constant units in order to understand the environmental properties of the planted crops. Crop production could be increased by better understanding the environmental properties of plantations and selecting crops suitable to the environment through this service.

## 4. Implementation and Results

To verify the agricultural environment monitoring server system proposed in this paper, a prototype was manufactured, installed in the real outdoors, and the system was tested. Even though the initial prototype version 1 could monitor all the outdoors environmental data such as soil information, environmental information, image information, location information acquiring from sensors and other physical devices through the GUI, it could not be used normally because the power consumed by the server was significantly larger than the power generated by the solar cells so there was a problem that making the effective use time short. In order to solve such a problem, the prototype version 2 includes a low-power system with embedded board modules, and incorporates a compact web server and database using built using Window CE-based mobile programming, so the power supply problem could be solved, and the outdoors environment could be monitored effectively.

### 4.1. System Configuration of Prototype Version 1

**Figure 6.**
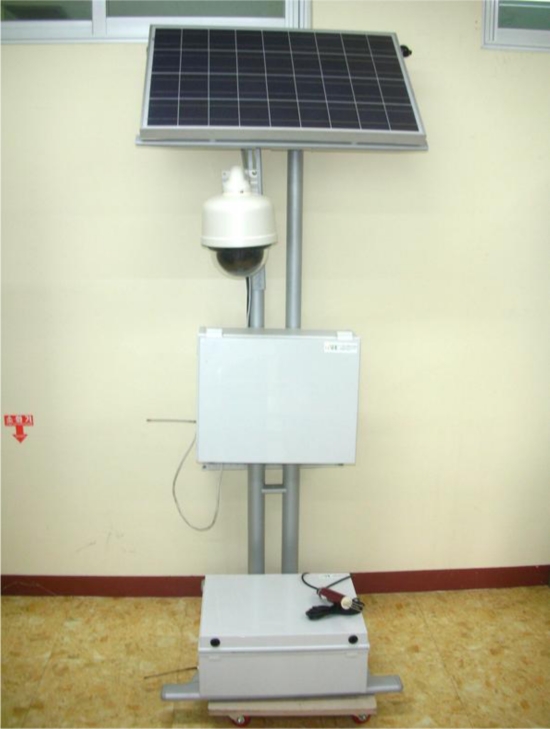
Prototype Version 1 of the Agricultural Environment Monitoring Server System.

The initially manufactured prototype version 1 of the agricultural environment monitoring server system is shown in Figure 6, and comprised soil sensors, environmental sensors, CCTV, and GPS module to collect information on the outdoors environment and image information, solar cells to supply power for the server system, and a server to process and store the collected information.

**Figure 7.**
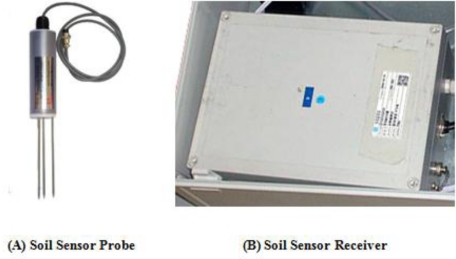
Soil sensor.

The soil sensors collect information on soil temperature and soil moisture in the outdoors environment, and Figure 7 and Table 1 show that the specifications of the soil sensors and the specific hardware used in the agricultural environment monitoring server system.

**Figure 8.**
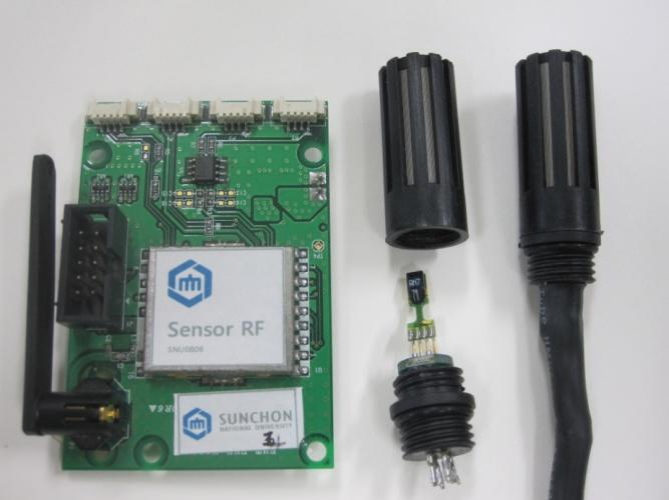
Environmental Sensor.

The environmental sensor node receives the sensor data from temperature, humidity, luminance sensors. It processes the data using a MSP430 MCU [27] and transmits them to relay nodes and gateways, using a CC2420 RF chip [28]. In order to reduce the impact of the heat the sensor receives from the node, the node and the sensor are kept at a certain distance from each other. The MSP430 is a 16 bit RISC with 48 Kbyte program memory and 10 Kbyte RAM inside. It can process multiple sensor data at high speed. CC2420 is a RF chip supporting Zigbee. It supports the 2,400 ∼ 2,483.5 MHz frequency band. It operates in DDDS method, supports the O-QPSK modulation method and 250 kbps baud rate. It enables real time wireless communication with small power consumption. Figure 8 and Table 2 show that the specifications of the environmental sensor and the hardware used in the agricultural environment monitoring server system.

For the CCTV an IP-based surveillance camera was installed. These cameras are normally used to determine when a burglary or an accident *etc.* has occurred, or to check the current outdoors environment in real time by monitoring and recording the outdoors environment 24 hours a day.

**Figure 9.**
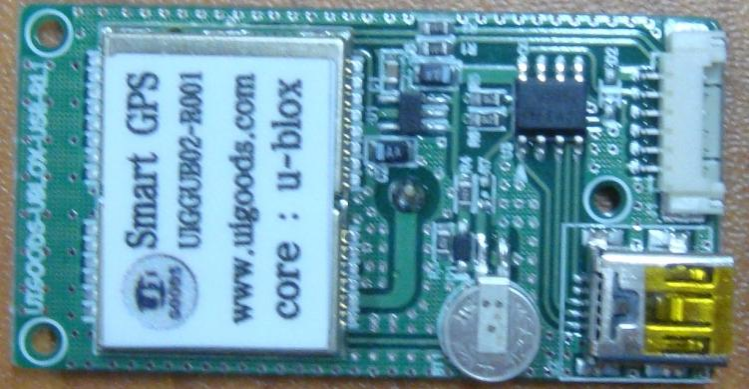
GPS Module.

The GPS module collects the location of the server system installed outdoors and the location information of the collected outdoors information. Figure 9 and Table 3 show that the specifications of GPS module and hardware used in the agricultural environment monitoring server system.

**Figure 10.**
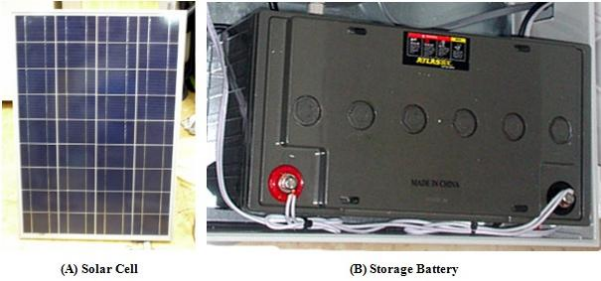
Solar Cell and Storage Battery.

The solar cell supplies power for the server system installed outdoors, and the server system could be operated through it even if no external power is supplied. Figure 10 shows the solar cells and batteries used in the agricultural environment monitoring server system.

**Figure 11.**
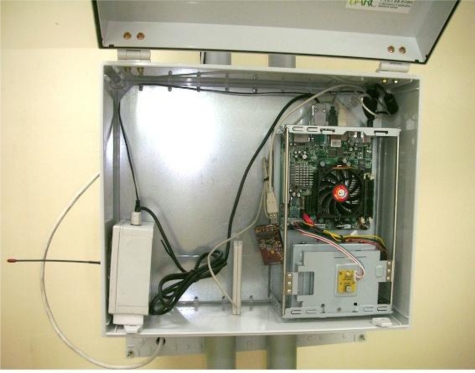
Agricultural Environment Monitoring Server (Database, Web Server).

The server processes and stores the collected information and provides it to users, and a database and a Web server are built on the low-power PC as seen in Figure 11.

Even though the prototype version 1 manufactured as described was shown to monitor environmental and soil information, image information, and location information acquiring from WSN sensors and other physical devices through the GUI, it has serious power consumption issues that limited its practical use.

### 4.2. System Configuration of Prototype Version 2

**Figure 12.**
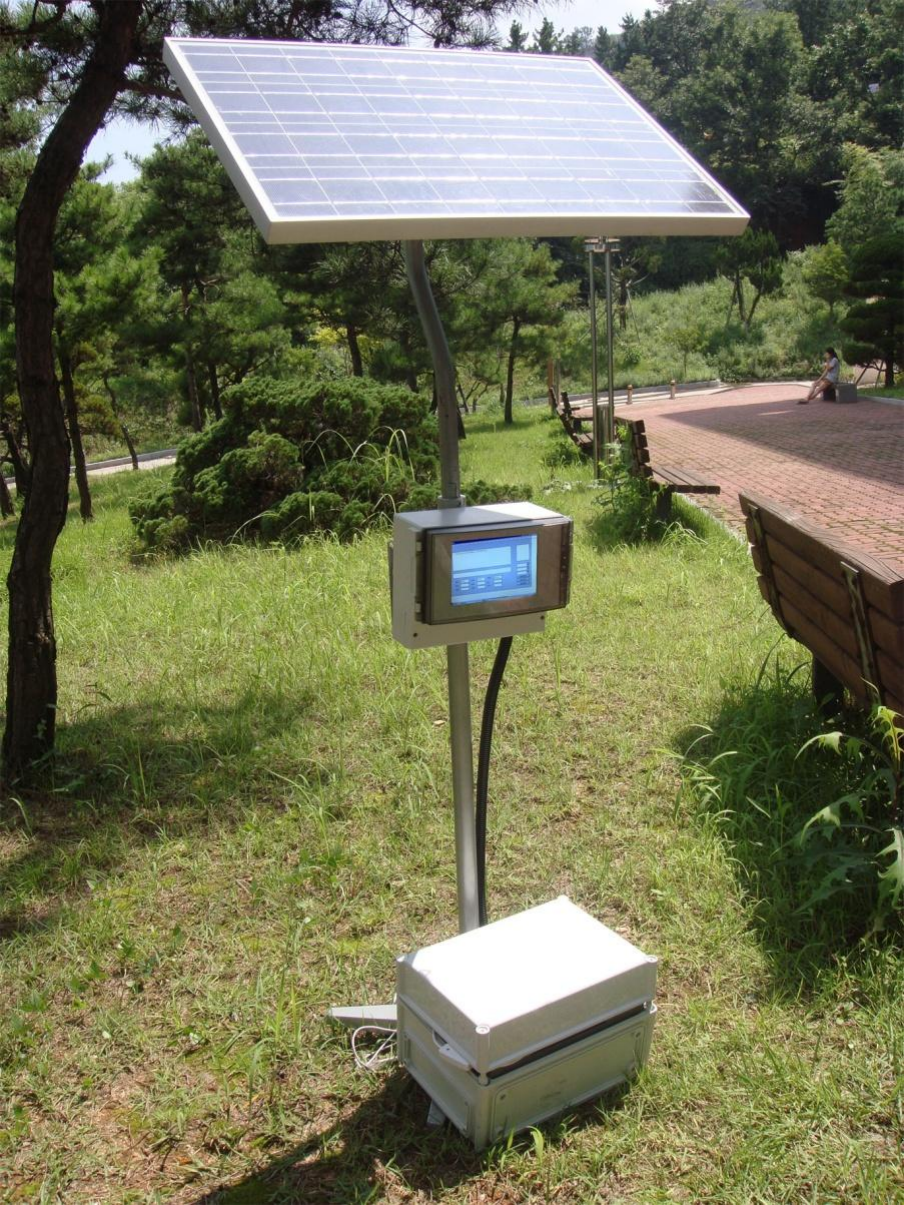
Prototype Version 2 of Agricultural Environment Monitoring Server System.

**Figure 13.**
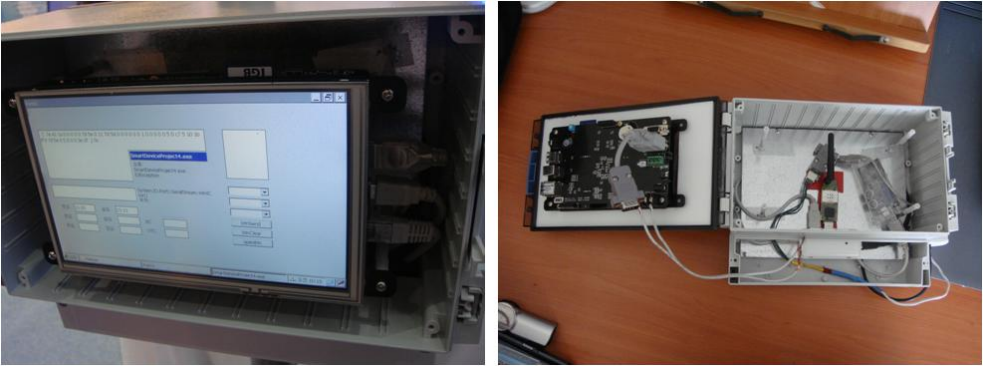
Embedded Board Module (Gateway, Database, and Web Server).

In order to solve the problems of prototype version 1, prototype version 2 was built to incorporate a server with low power embedded board modules, the CCTV camera was replaced to reduce power consumption, while the soil sensors, environmental sensors and GPS modules used were the same as in prototype version 1. Figure 12 shows the finished prototype version 2. Figure 13 shows the low power embedded board module used in prototype version 2, in which a database and a Web server are built to process and store the collected information or to provide it to users from the embedded board. The low power embedded board module used in prototype version 2 has the hardware specifications listed in Table 4.

The problems of prototype version 1 could be satisfactorily solved through these changes, and the agricultural environment monitoring server system could collect information without malfunctioning even in a real outdoors environment and provide real-time monitoring and various application services based on the information. Table 5 shows the power consumption of each module and the power supply of the solar cells installed on prototype version 2.

### 4.3. System Implementation

#### 4.3.1. Sensor Manager

**Figure 14.**
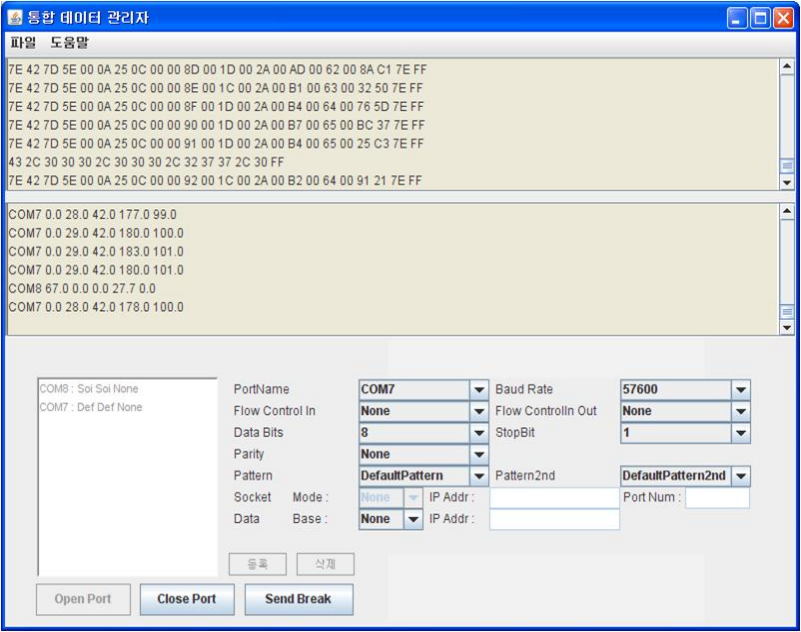
Implementation of the Sensor Manager.

The sensor manager in the proposed system processes the sensing data collected from the WSN sensor nodes installed outdoors to store in the database or to send the data to some other server system, and the implemented sensor manager parses the raw data collected from environmental and soil sensors into the required unit, as shown in Figure 14.

**Figure 15.**
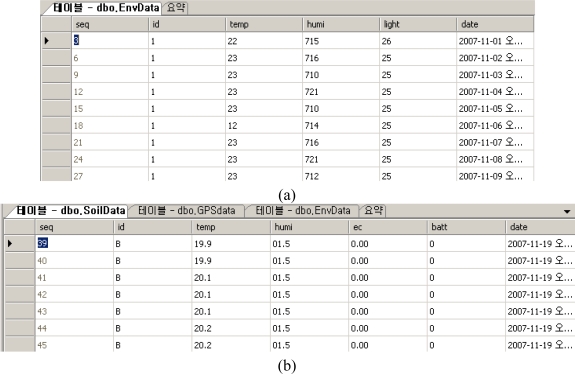
Environmental Information Table **(a)** and Soil Information Table **(b)**.

The soil and weather data values obtained by parsing the raw data are stored in the database through the sensor manager. Figure 15 shows the results of that the soil and weather data values obtained by parsing the raw data that are stored in the database.

**Figure 16.**
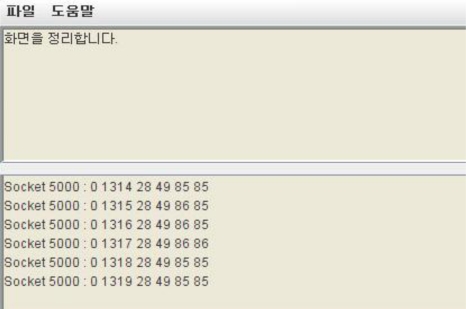
Screen that Receives Data Sent by Another Server System.

In addition, the sensor manager sends data to other server systems. Figure 16 is a screen showing the data sent by another server system, where environmental data values processed on another server are coming in, even though it does not collect any data through WSN sensors.

#### 4.3.2. GPS Manager

**Figure 17.**
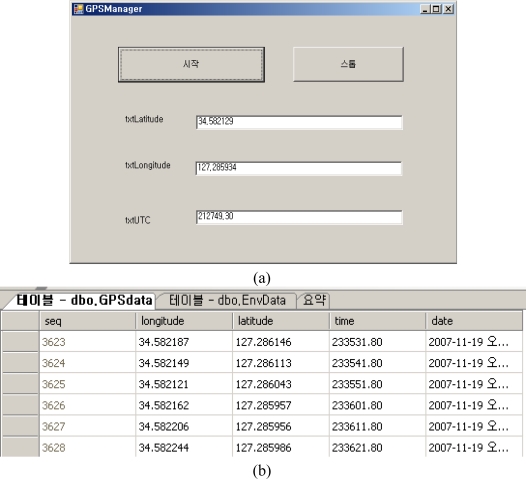
GPS Manager and GPS Information Table. **(a)** GPS Manager; **(b)** Location Information Table.

The GPS manager extracts location data coming in through serial communication from the GPS module to store it into the database. Figure 17 shows the location information manager interface and the location information values stored in the database.

#### 4.3.3. GUI

**Figure 18.**
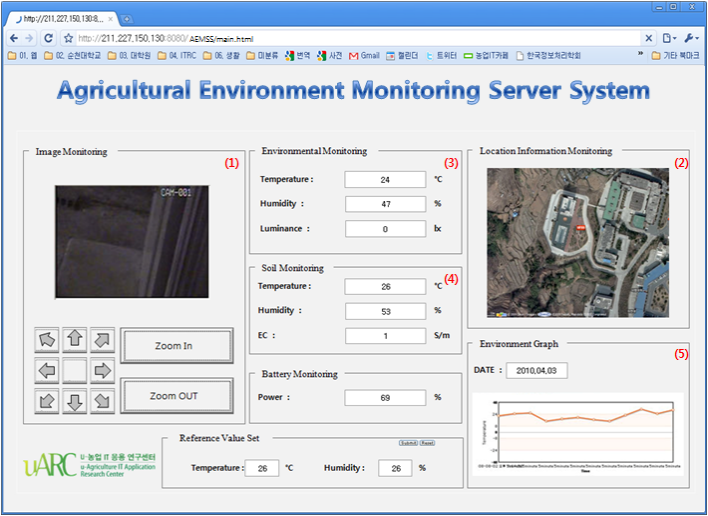
Web GUI for Agricultural Environment Monitoring Server System.

The data collected from the physical layer is processed by each manager for storage in the database, and is presented on the GUI to users via the Web server. Figure 18 below shows a Web GUI provided to users, where ① is a result of monitoring images, where the image data coming in through the camera is displayed as images of the place where this server is installed by the streaming service through the information manager. ② is a result of the location monitoring service, which shows that the current location data of the collected server system is mapped on the map with red markers. ③ and ④ display environmental and soil data stored in the database through the sensor manager. Finally, ⑤ shows data acquired from environmental sensors as a graphic form by averaging them.

From the implementation results described above, it could be seen that outdoors environmental information and image information could be collected through sensors and image surveillance cameras, and the outdoors environment could be always monitored through a user-intuitive GUI.

### 4.4. Field Test Results

To implement the design of the system proposed in this paper and evaluate its performance, the agricultural environment monitoring server system was installed at a real outdoor location to monitor the environment.

**Figure 19.**
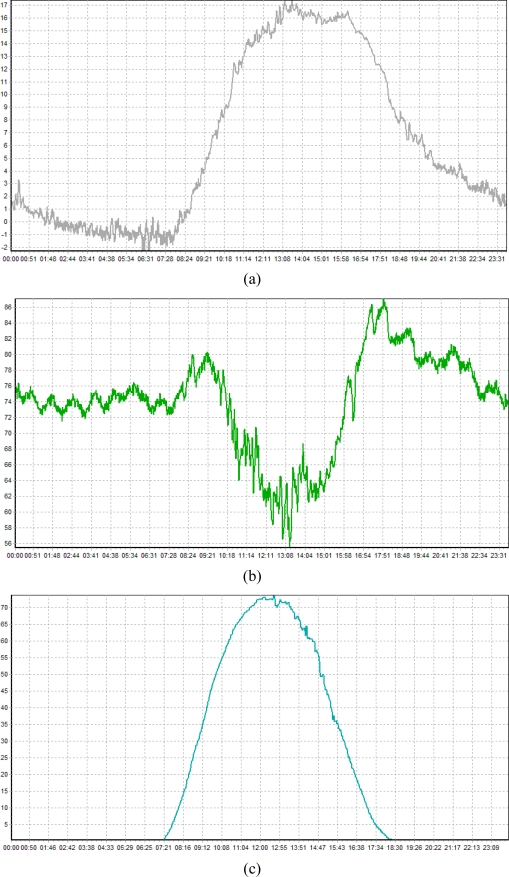
Measured Environmental Data Graph. **(a)** Temperature Graph (2010.02.23); **(b)** Humidity Graph (2010.02.23); **(c)** Luminance Graph (2010.02.23).

Figure 19 is a graph that represents environmental data measured by the proposed agricultural environment monitoring server system that is installed to be operational in the real agricultural environment, and it could be seen from this graph that the agricultural environment monitoring server system normally processes information sensed from WSN sensor nodes installed outdoors without malfunction.

**Figure 20.**
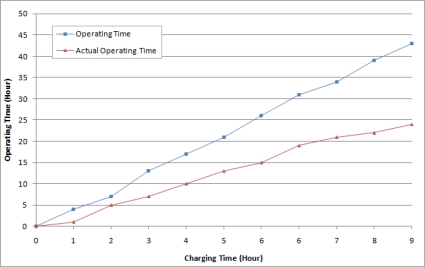
Field Test Result on Power Consumption of the Proposed System.

In addition, according to the power consumption field tests, the agricultural environment monitoring server system could be operated for about 24 hours by charging with the solar cells for about 10 hours, and the proposed system could be operated with only the solar cells installed on the system, without requiring a wired power supply connection or any additional charging process. Figure 20 is a graph that represents the results of the power consumption field test of the proposed system. Through the above results, it could be found that the proposed agricultural environment monitoring server system collects information even in the a real outdoors environment without malfunction and provides real-time monitoring and various application services based on the information.

## 5. Conclusions

This paper proposed an agricultural environment monitoring server system that integrates environmental and soil sensors, GPS module, CCTV *etc.* into a device to collect information for monitoring the environment at crop plantations, and provides real-time environmental monitoring and various application services based on the information. The proposed agricultural environment monitoring server system could work without supplying any external power in an agricultural environment with insufficient power infrastructure by using a solar cell module as its power supply.

Although a prototype of the agricultural environment monitoring server system was manufactured to monitor the outdoors environment for implementation of the designed system and performance evaluation, and the initial prototype version 1 could monitor the outdoors environment, however, it could not be used as planned because the power consumed by the server was significantly larger than the power generated in the solar cell module so that use time was short. In order to solve this problem, in prototype version 2 the server was rebuilt to implement the system with low power embedded board modules, and it was found that this version operated normally through the field tests.

If the agricultural environment monitoring server system proposed in this paper is applied to the agricultural environment, environmental information could be monitored even at a remote site, and it is expected that it would contribute to an increase in crop yields and the improvement of quality in the agricultural field by supporting the decision making of producers through the analysis of the collected information. Future study is scheduled to miniaturize the current system, and improve the current multi-server communication with socket communications to provide a structure in which communication is possible even in regions where the Internet is not connected.

## Figures and Tables

**Table 1.** Hardware Specifications of the Soil Sensor.

**Type**	**Specifications**
Measuring Range	Moisture: 0 ∼ 99.9% Temperature: 0 ∼ 60 °C
Accuracy	Moisture: ±3% Temperature: ±0.5 °C
Sensor Type	FDR (Frequency Domain Reflectometry)
Power Supply	DC 9 ∼ 15 [V]
Operating Range	0 ∼ 60 °C
Size	Probe length 12 cm 38 mm
Current	25 mA

**Table 2.** Hardware Specifications of the Environmental Sensors.

**Hardware Type**	**Hardware Specifications**
Processor (MSP430F1611)	Data Bus Width: 16 bit Program Memory Size: 48 KB Data RAM Size: 10 KB Maximum Clock Frequency: 8 MHz On-Chip ADC: 8-chx12-bit On-Chip DAC: 2-chx12-bit Number of Programmable I/Os: 48 Interface Type: USART Operating Supply Voltage: 1.8 V to 3.6 V Maximum Operating Temperature: 85 °C Minimum Operating Temperature: −40 °C
RF Device (CC2420)	Radio Frequency (Mhz): 2,400 Max. Data Rate (kbits/sec): 250 Antenna: PCB Antenna or SMA
Temperature Humidity Sensor (SHT-71)	Vmax (VDD): 2.4–5.5 Humidity range: 0–100% RH Humidity Accuracy: ±3% RH (20–80% RH) Repeatability: ±0.1% RH Temperature Accuracy: ±0.4 °C @ 25 °C
Luminance Sensor (GL5547)	Vmax (VDC): 150 Pmax (mW): 100 Ambient Temp (°C): ×30 ∼ +70 Spectral Peak (nm): 540 Response Time (ms): Rise 20, Decay 30

**Table 3.** Hardware Specifications of the GPS Module.

**Type**	**Specifications**
Max. Update	4 Hz
Sensitivity	Acquisition: −160 dBm Tracking: −160 dBm Cold starts: −160 dBm
Operating Temperature	−40 °C∼60 °C
Protocol	NMAEA

**Table 4.** Hardware Specifications of the Embedded Board Module.

**Type**	**Specifications**
System	CPU: 32 Bit RISC ARM1179JZF–667 MHz RAM: 128 MB(mDDR), OS: 48 MB/APP: 80 MB Flash: NAND Flash: 128 MB (OS: 50 MB, Storage: 78 MB) SD Memory: SD Support 16 GB Audio: Stereo Sound Power: DC 9 ∼ 24 V (9 V/500 mA) Operating Temperature: −10 ∼ 60 °C
Communication	RS232: 2 ch RS485: 1 ch (Auto) TTL: 1 ch USB Host: 2 ch–USB 1.1 USB Device: 1 ch–USB 2.0 Ethernet: 10 Mbps WLAN: USB Type Wireless LAN I2C: 1 ch SPI: 1 ch
Extension Port	GPIO: 16EA(3.3 V) PWM: 2 ch AD Converter: 12 Bit/4 ch

**Table 5.** Power Consumption of Module and Power Supply of Solar Battery.

**Module**	**Power consumption**		
	**Voltage**	**Current**	**Power**
GPS Module	DC 5 V	0.2 A	1 W
Embedded Board Module	DC 9 V	500 mA	5 W
CCTV Camera	DC 9 V	400 mA	4 W
Soil Sensor	DC 5 V	10 mA	0.05 W
Environmental Sensor	DC 3 V	2.3 A	6.9 W
**Total**	DC 12 V	1.4 A	16.95 W
**Module**	**Supply Power**		
	**Voltage**	**Current**	**Power**
Solar Cell	DC 26.4 V	7.6 A	200 W
Battery	**Voltage**	**Capacity (20HR)**	
	DC 12 V	64 A	

## References

[1] Kim MK, Park JH, Cho YW (2010). Current Trends and Industrial Strategies of IT Convergence.

[2] Lee KH, Ahn CM, Park GM (2008). Characteristics of the Convergence among Traditional Industries and IT Industry.

[3] Gim B-G, Lee W-J, Heo S-Y Construction of a Testbed for Ubiquitous Plant Factory Monitoring System Using Artificial Lighting.

[4] Shin Y-S (2006). A Study on Informatization Model for Agriculture in Ubiquitous Era.

[5] Park D-H, Kang B-J, Cho K-R, Sin C-S, Cho S-E, Park J-W, Yang W-M (2009). A Study on Greenhouse Automatic Control System Based on Wireless Sensor Network. Wireless Pers Commun.

[6] Jeong B-M (2006). Foreign u-Farm Service Model Casebook.

[7] Kwon OB, Kim J-H (2007). A Basic Direction for Building Agricultural Radio Frequency Identification Logistics Information System.

[8] Lee M-H, Shin C-S, Jo Y-Y, Yoe H (2009). Implementation of Green House Integrated Management System in Ubiquitous Agricultural Environments. J KIISE.

[9] Yoo N, Song G, Yoo J, Yang S, Son C, Koh J, Kim W (2009). Design and Implementation of the Management System of Cultivation and Tracking for Agricultural Products using USN. J KIISE.

[10] UDFC ALERT System Real-Time Flood Detection & Current Weather Conditions.

[11] Pierce FJ, Elliott TV (2008). Regional and on-Farm Wireless Sensor Networks for Agricultural Systems in Eastern Washington. Comput Electron Agric.

[12] Ayday C, Safak S Application of Wireless Sensor Networks with GIS on the Soil Moisture Distribution Mapping.

[13] Han W, Zhang N, Zhang Y A two-layer Wireless Sensor Network for Remote Sediment Monitoring.

[14] Hamrita TK, Hoffacker EC (2005). Development of a “Smart” Wireless soil Monitoring Sensor Prototype Using RFID Technology. Appl Eng Agric.

[15] USC Precision Agriculture.

[16] Baggio A Wireless Sensor Networks in Precision Agriculture.

[17] Burrell J, Brooke T, Beckwith R (2004). Vineyard Computing: Sensors Networks in Agricultural Production. Lect Note Comput Sci.

[18] Beckwith R, Teibel D, Bowen P Report from the Field: Results from an Agricultural Wireless Sensor Network.

[19] Morais R, Fernandes MA, Matos SG, Serodio C, Ferreira P, Reis M (2008). A ZigBee Multipowered Wireless Acquisition Device for Remote Sensing Applications in Precision Viticulture. Comput Electron Agric.

[20] Liu G, Ying Y (2003). Application of Bluetooth Technology in Greenhouse Environment, Monitor and Control. J Zhejiang Univ Agric Life Sci.

[21] Gonda L, Cugnasca CE A Proposal of Greenhouse Control Using Wireless Sensor Networks.

[22] Yoo S, Kim J, Kim T, Ahn S, Sung J, Kim D A2S: Automated Agriculture System Based on WSN.

[23] Lea-Cox JD, Kantor G, Anhalt J, Ristvey A, Ross DS A Wireless Sensor Network for the Nursery and Greenhouse Industry.

[24] Liu H, Meng Z, Cui S A Wireless Sensor Network Prototype for Environmental Monitoring in Greenhouses.

[25] Zhou YM, Yang XL, Guo XS, Zhou MG, Wang LR A Design of Greenhouse Monitoring & Control System Based on ZigBee Wireless Sensor Network.

[26] Yang I-C, Chen S, Huang Y-I, Hsieh K-W, Chen C-T, Lu H-C, Chang C-L, Lin H-M, Chen Y-L, Chen C-C, Lo YM RFID-Integrated Multi-Functional Remote Sensing System for Seedling Production Management.

[27] MSP430 Mixed Signal Microcontroller.

[28] CC2420 24 GHz IEEE 802.15.4 / Zigbee RF Transceiver.

